# Which treatment strategy for irreparable rotator cuff tears is most cost‐effective? A Markov model‐based cost‐utility analysis comparing superior capsular reconstruction, lower trapezius tendon transfer, subacromial balloon spacer implantation and reverse shoulder arthroplasty

**DOI:** 10.1002/jeo2.70180

**Published:** 2025-02-13

**Authors:** Jacob F. Oeding, Kyle N. Kunze, Ayoosh Pareek, Kristian Samuelsson

**Affiliations:** ^1^ Department of Orthopaedics, Institute of Clinical Sciences, The Sahlgrenska Academy University of Gothenburg Gothenburg Sweden; ^2^ Department of Orthopaedic Surgery Hospital for Special Surgery New York New York USA

**Keywords:** cost‐effectiveness, irreparable rotator cuff tear, Markov model, reverse shoulder arthroplasty, superior capsular reconstruction

## Abstract

**Purpose:**

Treatment options for irreparable rotator cuff tears (IRCTs) remain controversial and include superior capsular reconstruction (SCR), lower trapezius tendon transfer (LTTT), subacromial balloon spacer (SABS), and reverse shoulder arthroplasty (RSA). Despite reports of positive treatment responses with all approaches, the relative clinical benefit in the context of associated cost has not been well delineated. The purpose of this study was to determine the most cost‐effective treatment strategy among SCR, LTTT, SABS, and RSA for patients with massive IRCTs.

**Methods:**

A Markov Chain Monte Carlo probabilistic model was developed to evaluate the outcomes and costs of 1000 simulated patients undergoing either SCR, LTTT, SABS, or RSA for massive IRCTs. Upfront costs, health utility values, and reoperation rates including revisions and conversion to RSA were derived from the published literature. Outcome measures included costs, quality‐adjusted life years (QALYs), and the incremental cost‐effectiveness ratio (ICER).

**Results:**

Mean total costs of SCR, RSA, LTTT, and SABS were $30,540 ± 5,770, $26,896 ± 5,622, $25,819 ± 4,325, and $16,412 ± 2,583, respectively. On average, total QALYs from SCR, RSA, LTTT, and SABS were 6.17 ± 0.53, 3.78 ± 0.38, 5.33 ± 0.49, and 5.59 ± 0.48. Overall, SCR was determined the preferred, most cost‐effective strategy in 60% of patients included in the microsimulation model, with SABS the optimal strategy in 31% of cases and LTTT the optimal strategy in 9% of cases.

**Conclusion:**

SCR was found to be the most cost‐effective treatment option for IRCTs based on the current microsimulation and probabilistic sensitivity analyses, although LTTT and SABS were also found to be cost‐effective in select patients. Given that this statistical model does not consider the unique experiences of individual patients, shared decision‐making remains an important component in determining the optimal treatment strategy for IRCTs.

**Level of Evidence:**

Level III, economic decision model.

**Abbreviations**
ASES: American Shoulder and Elbow Surgeons
CMS: Constant‐Murley score
ICER: incremental cost‐effectiveness ratio
IRCT: irreparable rotator cuff tear
LTTT: lower trapezius tendon transfer
NMB: net monetary benefit
PSA: probabilistic sensitivity analysis
QALYs: quality‐adjusted life years
QOL: quality of life
RSA: reverse shoulder arthroplasty
SABS: subacromial balloon spacer
SCR: superior capsular reconstruction
WTP: willingness‐to‐pay

## INTRODUCTION

The management of irreparable rotator cuff tears (IRCTs) continues to be surrounded by substantial controversy, with no clear consensus regarding their optimal treatment strategy [9, 11, 27]. Management of these tears have been further complicated in recent years by an aging population with associated sarcopenia and inferior tissue quality but with increasing activity levels and shoulder functional demands [11]. Thus, while reverse shoulder arthroplasty (RSA) had traditionally been considered the gold‐standard treatment strategy for IRCTs, particularly for older adults, a number of new strategies aimed at improving functional outcomes have arisen [22].

Superior capsular reconstruction (SCR), lower trapezius tendon transfer (LTTT), subacromial balloon spacer implantation (SABS), and RSA currently represent four of the most common surgical options for management of IRCTs [11]. The goal of these strategies is to reduce contact between the humeral head and the acromion, thus minimizing glenohumeral joint pressures, decreasing pain, and enabling the deltoid muscle to be retrained as the predominant driver of glenohumeral range of motion [11]. Certain patient‐ and surgeon‐specific factors, such as the presence of rotator cuff arthropathy and surgeon comfort or expertise with a specific procedure, may limit the selection of treatment for IRCTs; however, in circumstances when such factors do not preclude any one treatment, the existence of several different approaches can present a substantial challenge during shared decision‐making discussions regarding the optimal treatment strategy.

While a substantial number of studies investigating the relative efficacy of these procedures has accumulated in recent years, no study to date has directly compared the cost‐effectiveness of these procedures. Thus, while patient‐ and surgeon‐specific factors often serve as primary determinants of treatment decisions and may play an important role in determining long‐term costs and outcomes associated with a variety of management options for IRCTs, understanding the relative cost associated with each procedure when several of the aforementioned treatments may be clinically appropriate may aid in decision‐making. Furthermore, given the increasing emphasis placed on value‐based care worldwide, decision‐making at the policy level will increasingly entail consideration of an intervention's demonstrated value, rather than outcomes alone.

Thus, the purpose of this study was to compare the cost‐effectiveness of SCR, LTTT, SABS, and RSA to determine the preferred cost‐effective treatment strategy for IRCTs. The authors hypothesized that RSA would prove to be the most cost‐effective treatment option for patients presenting with massive IRCTs.

## METHODS

### Markov modelling process

Markov models are decision trees used to model clinical courses of treatment as transitions between discrete health states based on probabilistic events occurring over a specified period of time [2]. As summarized below, each state is associated with a quality‐of‐life (QOL) value, transition probabilities, and costs that are determined from empirically‐derived or estimated data. QOL values range from 0 (death) to 1 (perfect health) and provide a measure of disease burden on an individual's life. According to the transition probabilities, experimental patients transition between health states through Markov cycles, accruing QOL values and costs at each state.

QOL values are also aggregated into quality‐adjusted life‐years (QALYs). The primary outcome of a Markov study is the incremental cost‐effectiveness ratio (ICER), which is defined as the difference in costs between two treatment options divided by the difference in QALYs for those treatments [17]. For the present study, treatment strategies were (1) SCR, (2) LTTT, 3) SABS, and 4) RSA. A willingness‐to‐pay (WTP) threshold is set and defined as the maximum amount that society is willing to pay to achieve one additional QALY. A WTP of $50,000 is considered standard [25]. This threshold has been widely accepted and applied across a range of similar economic analyses in orthopaedics [23, 26, 30, 31]. If a treatment results in an ICER below the WTP threshold, it is considered cost‐effective; as a result, the treatment with the most QALYs (lowest ICER) would be considered the optimal strategy. If a treatment results in both lower costs and more QALYs, it is considered to be a ‘dominant treatment’ [6].

### Model structure

**Figure 1:**
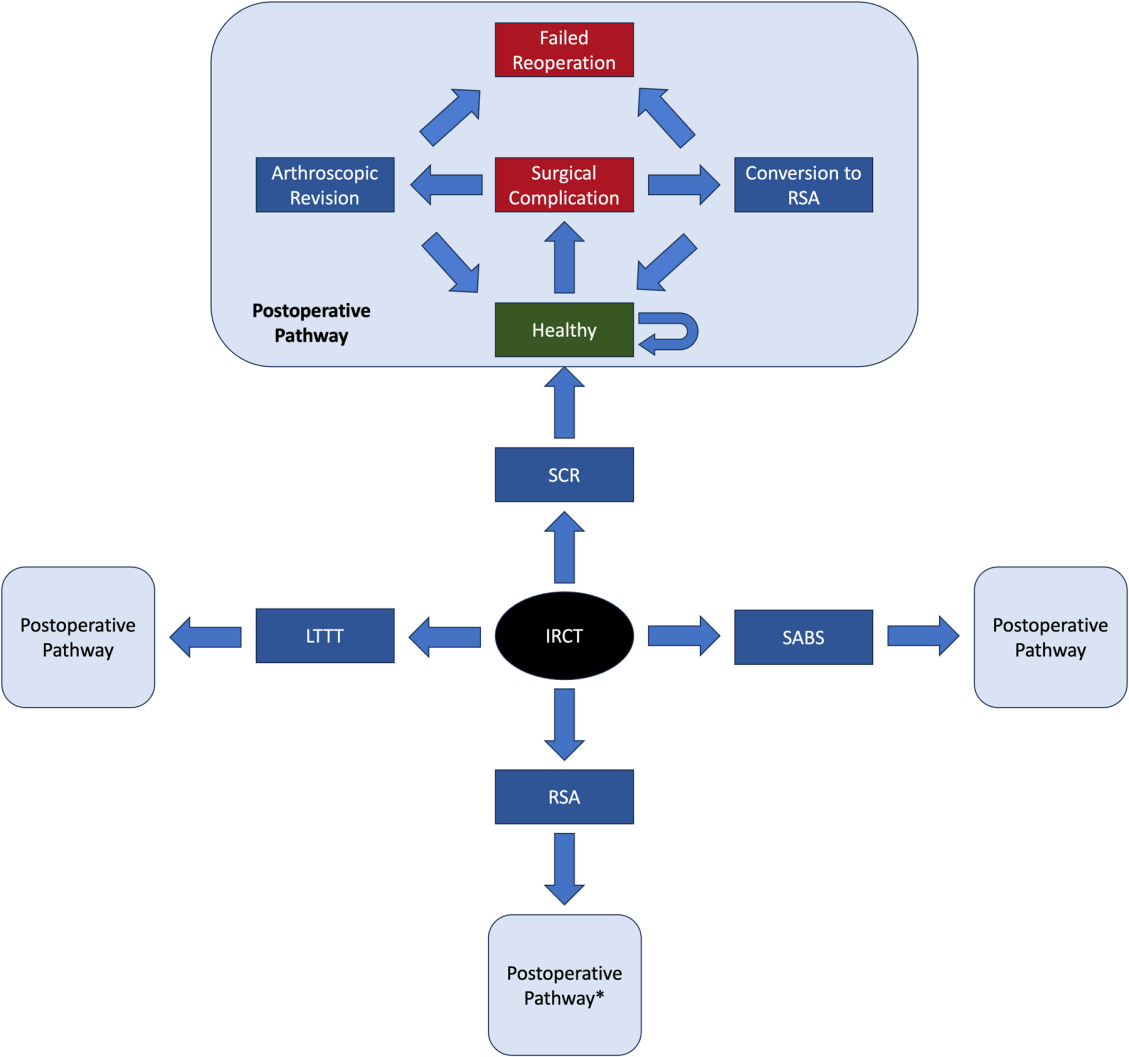
Markov model diagram depicting flow of patients in the decision model. *Patients experiencing a surgical complication after RSA are assumed to undergo revision RSA. IRCT, irreparable rotator cuff tear; LTTT, lower trapezius tendon transfer; RSA, reverse shoulder arthroplasty; SABS, subacromial balloon spacer; SCR, superior capsular reconstruction.

The Markov decision tree model utilized in the current study was constructed from publicly available software (TreeAge Pro; TreeAge Software). A patient presenting with a torn and irreparable supraspinatus tendon (with or without a torn and irreparable infraspinatus tendon) as well as a torn and reparable subscapularis tendon that has failed to respond to 6 months of nonoperative therapy serves as the base case for our model. This patient represents the demographic most likely to be faced with the decision between SCR, LTTT, SABS, and RSA and most closely reflects the cohort of patients included in studies from which model inputs were derived. All patients in the model are assumed to have a symptomatic massive IRCT with no glenohumeral arthropathy, infections, allergies to polylactide or polycaprolactone, or axillary nerve palsy at the time of presentation. Following each index surgical procedure, patients follow the postoperative pathway as depicted in Figure 1. Probabilistic subsets of patients experiencing a surgical complication after SCR, LTTT, or SABS are assumed to undergo either conversion to RSA or arthroscopic revision based on probabilities derived from the literature, while patients who experience a surgical complication after RSA are assumed to undergo revision RSA.

### Model parameters

To obtain outcomes and model inputs for patients with IRCTs treated with SCR, LTTT, SABS, and RSA, a targeted literature search was performed. Reoperation rates including conversion to RSA as well as revision RSA after a primary RSA were derived from six recent systematic reviews evaluating outcomes after management of IRCTs with each surgical option (Table 1) [3, 10, 19, 20, 24, 29].

**Table 1:** Model inputs.

	Base case value	PSA 10%–90% range (distribution type)	Source
*Transition probabilities*			
Reoperation after SCR	0.069	0.051–0.087 (*β*)	Smith et al. [29]
Conversion to RSA After SCR	0.017	0.013–0.021 (*β*)	Mercurio et al. [24]
Reoperation after LTTT	0.075	0.056–0.096 (*β*)	de Marinis et al. [10]
Conversion to RSA After LTTT	0.05	0.037–0.064 (*β*)	de Marinis et al. [10]
Reoperation after SABS	0.069	0.051–0.088 (*β*)	Levy et al. [20]
Conversion to RSA after SABS	0.05	0.037–0.063 (*β*)	Berk et al. [3]
Revision RSA	0.08	0.061–0.099 (*β*)	Kovacevic et al. [19]
*Costs (US $)*			
SCR	$20,837	$15,546–$26,343 (*γ*)	Marigi et al. [22], Institutional Data
LTTT	$16,915	$12,754–$21,581 (*γ*)	Marigi et al. [22], Institutional Data
SABS	$9,058	$6,894–$11,487 (*γ*)	Luthringer et al. [21], Institutional Data
RSA	$17,210	$12,996–$22,041 (*γ*)	Marigi et al. [22], Institutional Data
*Utilities*			
Success after SCR	0.76	0.69–0.82 (normal)	Mercurio et al. [24]
Success after LTTT	0.67	0.61–0.74 (normal)	de Marinis et al. [10]
Success after SABS	0.69	0.62–0.75 (normal)	Berk et al. [3]
Success after RSA	0.59	0.52–0.65 (normal)	Kovacevic et al. [19]
Failure	0.35	0.28–0.42 (normal)	Berk et al. [3]

Abbreviations: LTTT, lower trapezius tendon transfer; RSA, reverse shoulder arthroplasty; SABS, subacromial balloon spacer; SCR, superior capsular reconstruction.

Costs were considered from the payer perspective (commercial or government). Costs for each treatment strategy and transition state in the model were derived from the published literature and verified against actual patients’ costs at our institution (a large, academic medical centre) for accuracy and reliability (Table 1) [21, 22]. The cost for arthroscopic revisions were assumed to be 5% greater than their primary counterparts based on prior cost‐effectiveness analyses [13, 18].

The Constant‐Murley score (CMS) was used to quantify the utility of each treatment strategy based on the outcomes of four recent systematic reviews on SCR, LTTT, SABS, and RSA performed in patients with IRCTs (Table 1) [3, 10, 19, 24]. The CMS was selected, as it was the most commonly reported patient‐reported outcome measure among each of the four treatment strategies and provides an overall measure of shoulder function that can be used to approximate shoulder‐related quality of life [1]. To convert from the CMS 100‐point scale to the shoulder‐related QOL values used as model inputs, scores were scaled by a factor of 1/100 such that they ranged between 0 and 1.

### Monte Carlo microsimulation and probabilistic sensitivity analysis (PSA)

In contrast to methods used to create predictive models with fixed input values, Monte Carlo simulation enables the construction of models that leverage probability distributions for variables with inherent uncertainty. In the present study, Monte Carlo microsimulation was used to generate hypothetical patients who repeatedly traverse the model, each time with a set of different input parameters drawn from a corresponding probability distribution. For each microsimulation, patients accrue costs and utilities, and these are averaged and compared over many simulated cycles to produce more robust results that consider the uncertainty associated with estimated model inputs. A greater number of cycles that produce similar results corresponds to increased confidence that the result in question does in fact reflect reality despite the inherent uncertainty associated with selected input parameters.

In this study, PSA was used to simultaneously vary all cost, utility, and transition probability input parameters in the model. PSA has been shown to better estimate uncertainty in the model when compared to standard sensitivity analysis for microsimulation models [4, 14]. One thousand patients were simulated over 1,000 cycles, with (1) cost parameters assigned gamma distributions based on their means, (2) probability parameters assigned beta distributions based on their means, and (3) utilities assigned normal distributions based on their means [12]. Standard deviations for probabilities and costs were assumed to be 20% of the mean based on prior analyses [7]. For utility values, a standard deviation of 0.05 was assumed. In this study, both costs and QALYs were discounted at a rate of 3% annually [28]. The ICER was used to evaluate the cost‐effectiveness of each treatment arm by providing a measure of the cost per year acquired by undergoing the specific treatment arm that results in the highest number of QALYs. Cycle length was defined as one year, with an overall time‐horizon of 10 years.

## RESULTS

### Monte Carlo microsimulation and PSA

The results of the Monte Carlo microsimulation and PSA are shown in Table 2. Mean total costs of SCR, RSA, LTTT, and SABS were $30,540 ± 5,770, $26,896 ± 5,622, $25,819 ± 4,325, and $16,412 ± 2,583, respectively. On average, total QALYs from SCR, RSA, LTTT, and SABS were 6.17 ± 0.53, 3.78 ± 0.38, 5.33 ± 0.49, and 5.59 ± 0.48. Because SABS demonstrated both lower costs and greater or similar utility to LTTT and RSA, both LTTT and RSA were considered ‘absolute dominated’ treatment strategies. However, because SCR demonstrated greater utility than SABS, it was considered an ‘undominated’ treatment strategy despite its increased cost relative to SABS. In this case, when no treatment results in both lower costs and greater utility, the ICER is used to determine the most cost‐effective intervention at a given WTP threshold.

**Table 2:** Results of Monte Carlo microsimulation and PSA.

Dominance	Strategy	Cost ($)	Incremental cost ($)^a^	Effectiveness (QALYs)	Incremental effectiveness (QALYs)^a^	ICER ($/QALY)	NMB ($)^b^	% Iterations cost‐effective (%)
Undominated	SABS	16,412 ± 2583		5.59 ± 0.48			263,082 ± 24,628	31
Absolute dominated	LTTT	25,819 ± 4325	+9406	5.33 ± 0.49	−0.26	−36,247.55	240,701 ± 25,784	9
Absolute dominated	RSA	26,896 ± 5622	+10,483	3.78 ± 0.38	−1.81	−5782.28	161,947 ± 20,912	0
Undominated	SCR	30,540 ± 5770	+14,127	6.17 ± 0.53	+0.58	24,332.09	277,985 ± 27,986	60

Abbreviations: ICER, incremental cost‐effectiveness ratio; LTTT, lower trapezius tendon transfer; NMB, net monetary benefit; PSA, probabilistic sensitivity analysis; QALY, quality adjusted life year; RSA, reverse shoulder arthroplasty; SABS, subacromial balloon spacer; SCR, superior capsular reconstruction.

a Relative to SABS.

b The NMB represents the value of an intervention in monetary terms when a willingness to pay threshold for a unit of benefit (one QALY) is known. It is calculated as the benefit of a therapy expressed in monetary terms minus the costs associated with that therapy.

**Figure 2:**
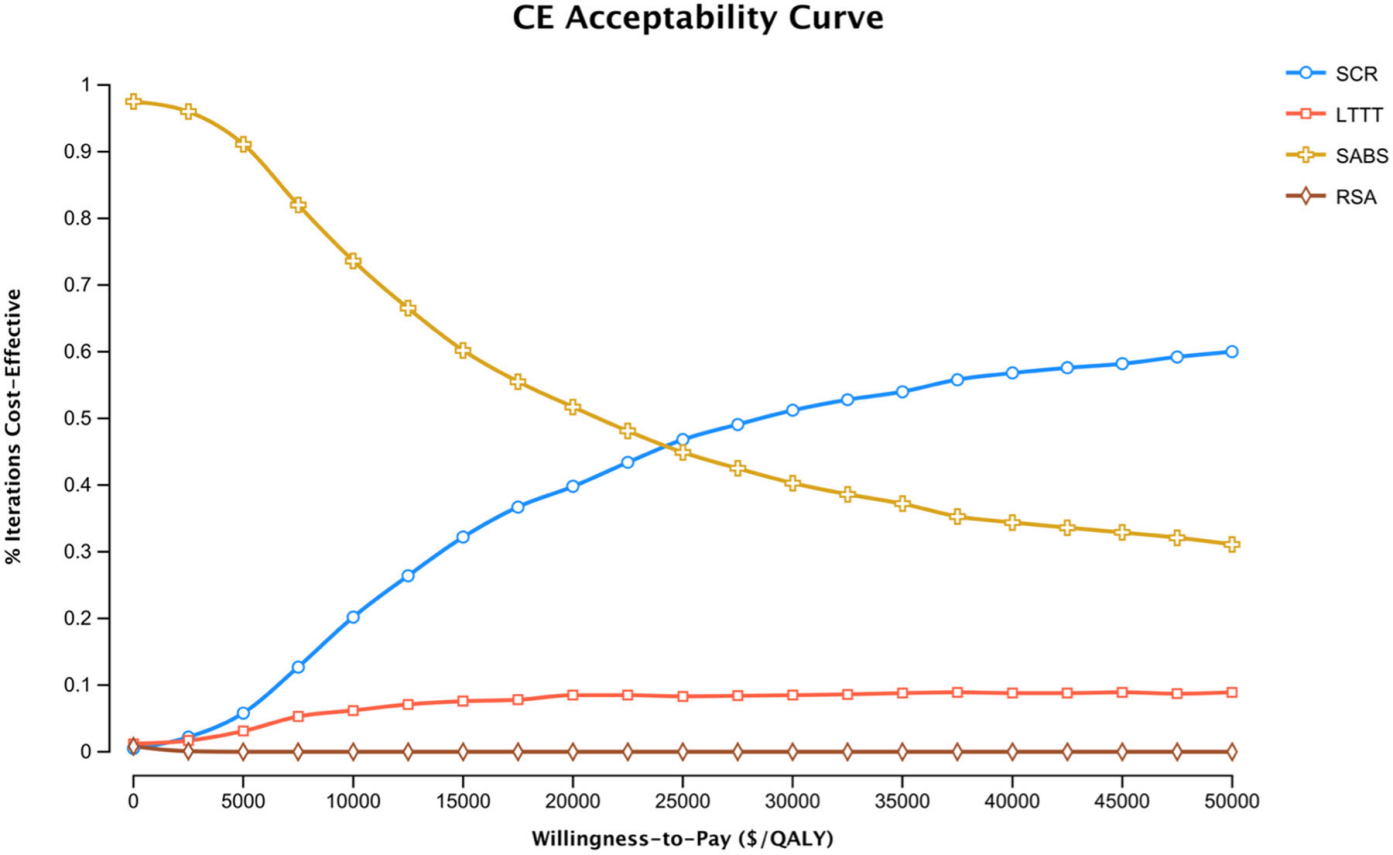
Cost‐effectiveness (CE) acceptability curves for each treatment strategy at willingness‐to‐pay thresholds from $0/QALY to $50,000/QALY. SCR, superior capsular reconstruction; LTTT, lower trapezius tendon transfer; QALY, quality adjusted life year; RSA, reverse shoulder arthroplasty; SABS, subacromial balloon spacer.

Assuming the standard $50,000 WTP threshold for cost‐effective interventions [25], SCR was determined to be the most cost‐effective treatment strategy, with an ICER of $24,332/QALY relative to SABS. Figure 2 displays cost‐effectiveness acceptability curves for each treatment strategy at WTP thresholds from $0/QALY to $50,000/QALY. The WTP threshold below which SABS became cost‐effective over SCR was found to be less than $25,000/QALY, suggesting that SCR is a highly cost‐effective treatment strategy, even at lower WTP thresholds.

**Figure 3:**
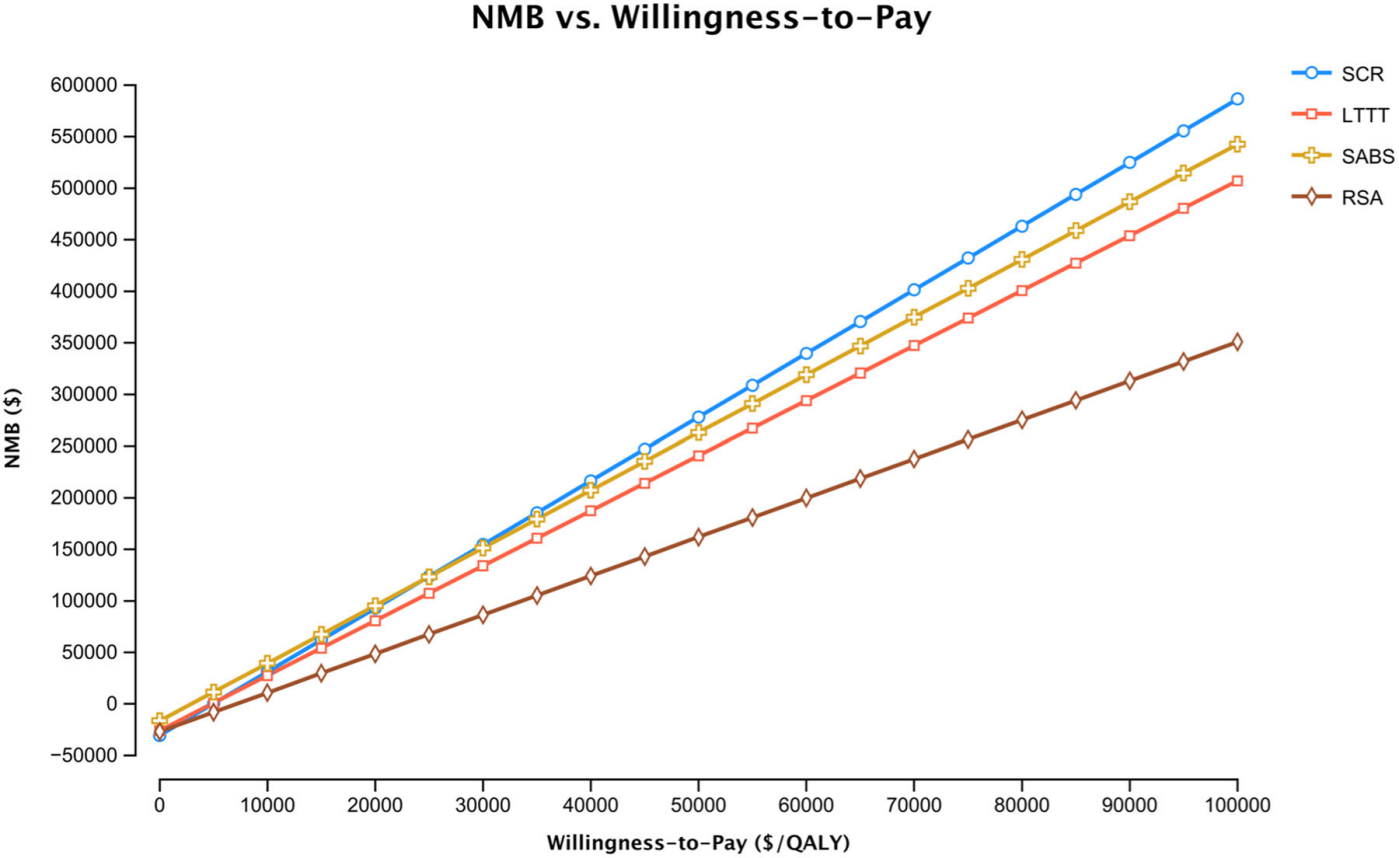
Net monetary benefit (NMB) of each treatment strategy at willingness‐to‐pay thresholds from $0/QALY to $100,000/QALY. LTTT, lower trapezius tendon transfer; QALY, quality adjusted life year; RSA, reverse shoulder arthroplasty; SABS, subacromial balloon spacer; SCR, superior capsular reconstruction.

Figure 3 shows the net monetary benefit (NMB) of each treatment strategy at WTP thresholds from $0/QALY to $100,000/QALY. The NMB represents the value of an intervention in monetary terms when a WTP threshold for a unit of benefit (one QALY) is known. It is calculated as the benefit of a therapy expressed in monetary terms minus the costs associated with that therapy.

**Figure 4:**
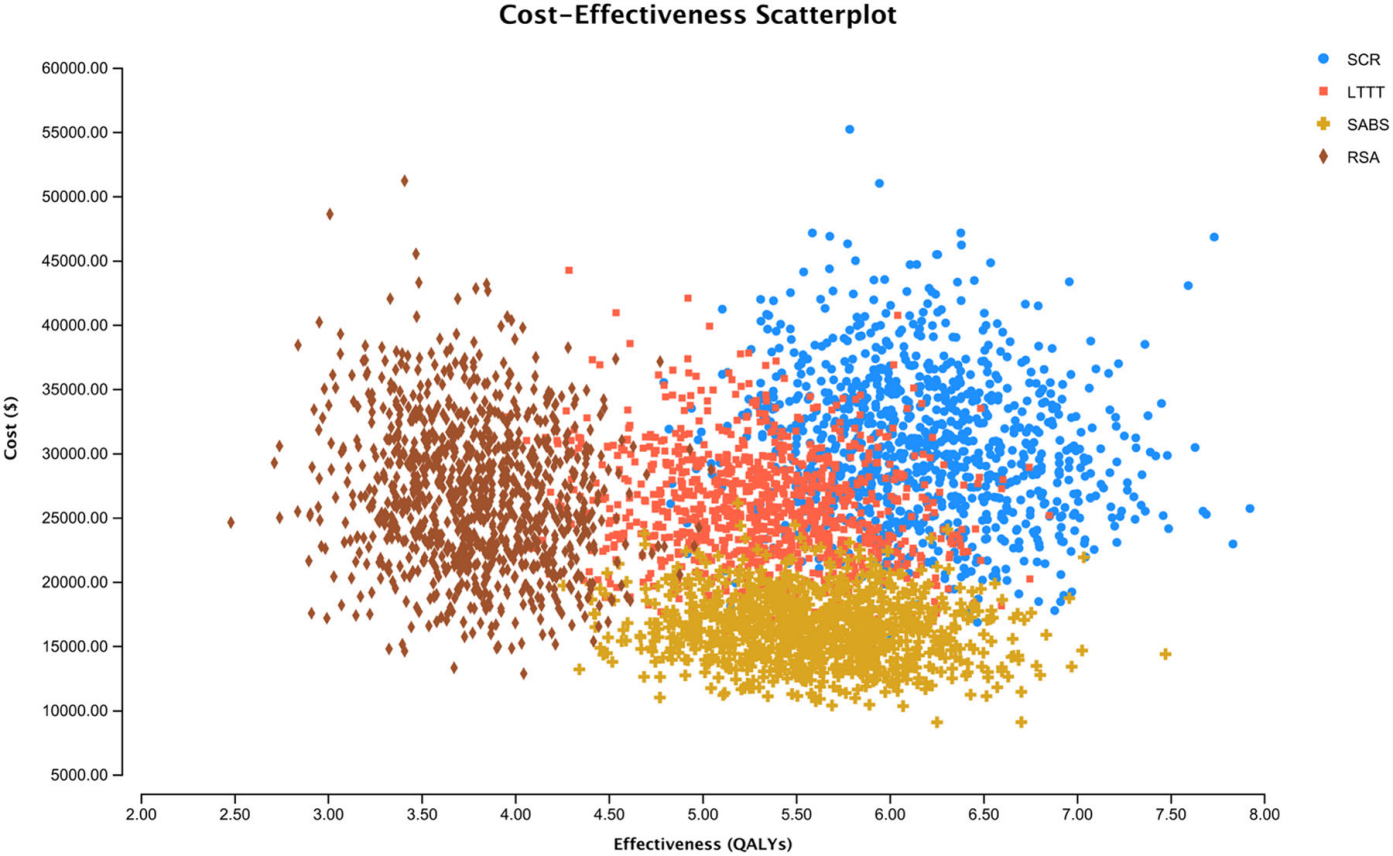
Costs and quality adjusted life years (quality adjusted life years [QALYs]) gained per patient for each of the 1000 patients in the Monte Carlo microsimulation model. LTTT, lower trapezius tendon transfer; RSA, reverse shoulder arthroplasty; SABS, subacromial balloon spacer; SCR, superior capsular reconstruction.

Results of the microsimulation and PSA are depicted in Figure 4. Out of 1000 samples run over 1000 trials, SCR was determined the preferred, most cost‐effective strategy for 60% of patients included in the microsimulation model, with SABS being the optimal strategy in 31% of cases, and LTTT the optimal strategy in the remaining 9% of cases.

**Figure 5:**
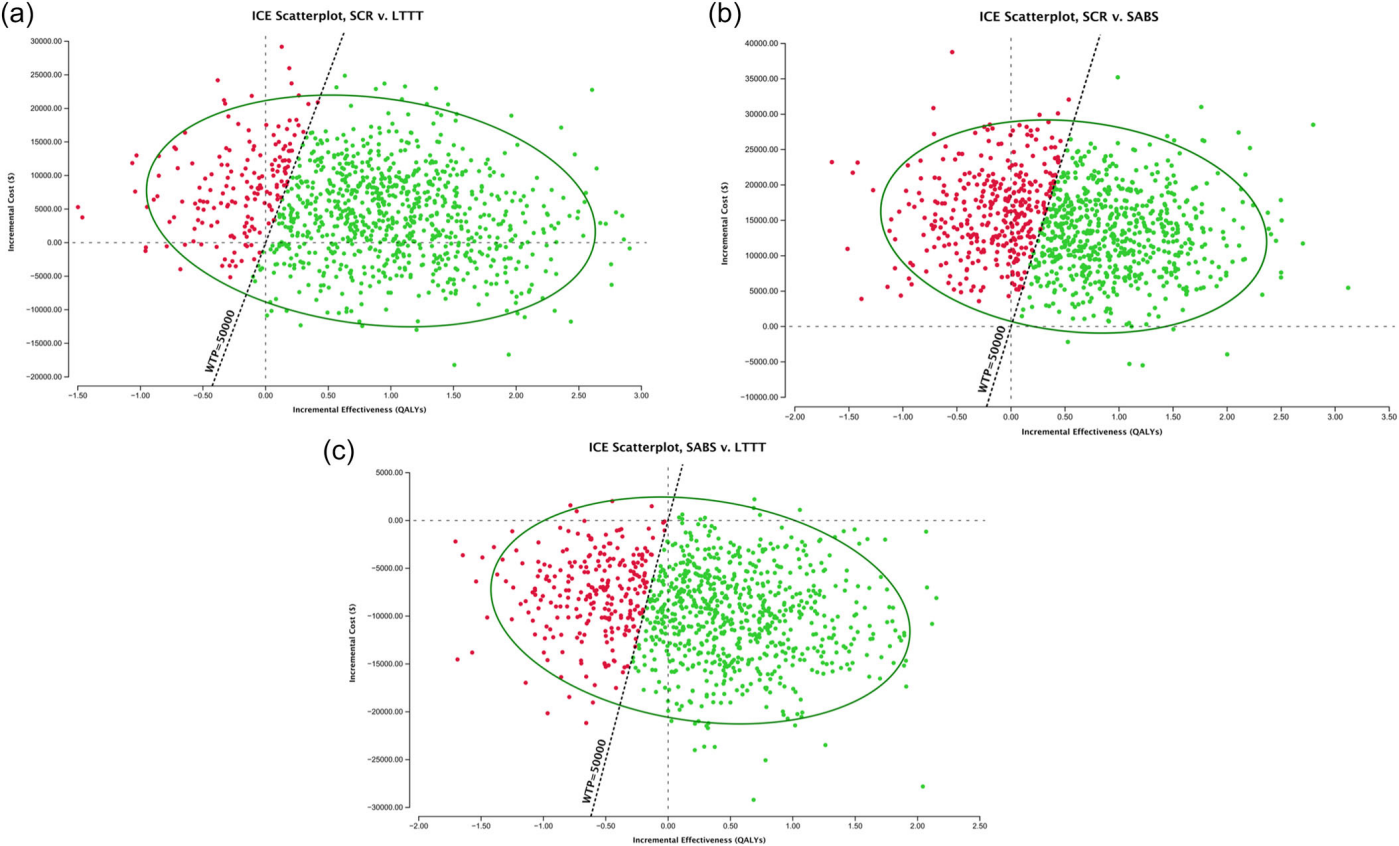
Incremental cost‐effectiveness scatterplots from the probabilistic sensitivity analysis (PSA), shown with 95% confidence ellipses. All points to the right of the diagonal willingness‐to‐pay (WTP) line represent patients for whom the model predicted superior capsular reconstruction (SCR) (a and b) or subacromial balloon spacer (SABS) (c) as more cost‐effective than lower trapezius tendon transfer (LTTT) (a and b) or SABS (b) at a WTP of $50,000/quality adjusted life years (QALYs) (green). Points to the left of the $50,000 WTP diagonal correspond to patients for whom LTTT (a and c) or SABS (b) is the optimal strategy (red), and points both below the $0 incremental cost line and to the right of the $50,000 WTP line correspond to patients for whom the points in green represent the dominant preferred treatment strategy.

The ICER scatterplots shown in Figure 5 illustrate the model's predictions and confidence for patients faced with the decision of SCR versus LTTT, SCR versus SABS, and SABS versus LTTT. Points in green correspond to patients for whom the model correctly predicted the most cost‐effective treatment strategy (SCR over LTTT, SCR over SABS, or SABS over LTTT), while points in red correspond to patients for whom the alternative strategy would have been the most cost‐effective treatment strategy. The model's confidence is shown with a 95% confidence ellipse.

## DISCUSSION

The primary findings of the current study were (1) SCR was determined to be the most cost‐effective treatment strategy for the management of IRCTs when compared to SABS, LTTT, and RSA, (2) SABS may be a cost‐effective alternative treatment option for the management of IRCTs when compared to LTTT and at low WTP thresholds, and (3) LTTT and RSA were found to be ‘absolute dominated’ treatment strategies, resulting in neither greater utility nor lower costs when compared to SABS.

As these results were determined across a range of procedural costs, they can be more broadly and confidently applied to different practice types and locations. A recent scoping review found extremely limited data published on the cost of SCR, with only two studies meeting the authors’ inclusion criteria [15]. They concluded that primary drivers of cost associated with SCR were related to intraoperative factors including implants, specifically human dermal allograft and anchors, as well as operative time. The authors argued that future studies not only reporting costs, but also the cost‐effectiveness of the procedure, were necessary to quantify whether the high costs of SCR can be justified by an associated improved utility [15]. Only a single‐institution analysis comparing SCR to RSA at short‐term follow‐up has reported some form of a related metric that attempted incorporate both costs and outcomes, which was defined as the change in the American Shoulder and Elbow Surgeons (ASES) score divided by total direct costs per $10,000 [16]. The authors were unable to demonstrate a statistically significant difference in value between RSA and SCR using this metric [16]. This could be explained by the single‐institution nature of the study, relatively small sample size with only 30 RSAs and 126 SCRs included for analysis, short‐term follow‐up of just one‐year, and the potential learning curve associated with SCR as a relatively new procedure [16]. The authors did report significant differences in both operating room time and complication rates between the two procedures, with SCR demonstrating a significantly longer operating room time (204 vs. 108 min) and significantly lower complication rate (3% vs. 13%) compared to RSA [16]. These findings would align with the results of our model demonstrating both greater costs (theoretically associated with longer operating room time) but also the greatest number of QALYs achieved (potential for fewer complications) with SCR, as operating room time has been shown to be one of the greatest contributors to the cost of SCR [15, 22].

The current study demonstrated that SABS may be a cost‐effective treatment strategy for IRCTs when performed in appropriately selected patients, with average costs totalling $16,412 ± 2583 and average QALYs totalling 5.59 ± 0.48. Importantly, while QALYs achieved with SABS were comparable to those of the other procedures, total costs were substantially lower. This finding aligns with two prior cost analyses of SABS that demonstrated substantially lower upfront costs with SABS relative to the other procedures included in this study: one study compared SABS to partial rotator cuff repair, RSA, and conservative management in the Italian healthcare system [5], while one compared SABS to partial rotator cuff repair in the United States [21]. This cost was validated against our own institutional cost data, which also confirmed substantially lower upfront costs with SABS relative to SCR, LTTT, and RSA. Because of these reduced upfront costs, likely due to reduced operating room time, SABS was able to produce moderate outcomes relative to the other procedures and remain cost‐effective. It is important to note, however, that prior work has shown SABS to be most beneficial for a relatively specific patient population with IRCTs, and thus the indications for SABS may be slightly different than those for SCR, LTTT, or RSA [8]. Indeed, ideal candidates for SABS are patients with IRCTs and an intact subscapularis with a chief complaint of pain, but who also present with a preserved range of motion [8]. Therefore, SABS may be beneficial in patients with medical comorbidities that would otherwise limit the use of other techniques dependent on biologic tissue healing (i.e., SCR, LTTT) or whose medical profile was such that the amount of time exposed to anaesthesia needs to be limited. However, poor candidates for SABS would include patients with pseudoparalysis, axillary nerve palsy, irreparable subscapularis tears, or severe glenohumeral arthritis (Hamada grade ≥ 3); [8] for these patients, SABS would likely prove not cost‐effective and other treatments included in this study should be considered.

Neither LTTT nor RSA demonstrated superior cost‐effectiveness when compared to the alternative treatment strategies considered in the model. These findings are likely explained by lower functional scores relative to SCR and increased costs relative to SABS. For example, a recent systematic review comparing patient‐reported outcomes after different treatment strategies for IRCTs found that patients who received RSA demonstrated an average CMS score of just 59 [19]. In comparison, the average CMS score for patients undergoing SCR was 76 [24]. This greater functionality translates to greater utility and provides one explanation for why SCR and LTTT were found to be more cost‐effective than RSA. However, it is again important to consider individual patient factors when deciding between RSA and other surgical treatment alternatives for IRCTs, as a number of patient‐specific factors and comorbidities that limit tissue healing may predispose some patients to experience greater cost‐utility with RSA relative to the other procedures included in the present model. In addition, it is important to note that all patients in the current model were assumed to have a symptomatic massive IRCT with *no* glenohumeral arthropathy. This likely biased the model to favour all treatments except RSA. If a different patient demographic, such as one *with* glenohumeral arthropathy, had been selected to serve as the base case for our model, outcomes would have likely differed. For example, in scenarios involving patients with glenohumeral arthropathy, it is likely that RSA would be determined to be one of the cost‐effective treatment options.

### Limitations

There are limitations to this study that should be noted to ensure interpretation of the results in their proper context. First, a Markov model is a rigid model for an ‘average’ patient undergoing a treatment and must define definitive likelihoods of treatment failure, costs, and treatment pathways. While this limitation is minimized when applying Monte Carlo microsimulation and PSA, our methodology did not capture the unique experience of every patient undergoing treatment for an IRCT. In practice, numerous factors that could not be captured in this model exist and may influence decision making. Second, to conduct the analysis, assumptions regarding transition probabilities and outcomes using the current literature were necessary. Finally, as multiple factors contribute to an individual patient's potential for tissue healing, implant integration, and quality of life, the current results may not be generalizable to all patients. This limitation is mitigated by performing sensitivity analyses that account for a spectrum of model inputs; however, it is not possible to remove all uncertainty from the analysis.

## CONCLUSION

SCR was found to be the most cost‐effective treatment option for IRCTs based on the current microsimulation and probabilistic sensitivity analyses, although LTTT and SABS were also found to be cost‐effective in select patients. Given that this statistical model does not consider the unique experiences of individual patients, shared decision‐making remains an important component in determining the optimal treatment strategy for IRCTs.

## AUTHOR CONTRIBUTIONS

*Conceptualization, data procurement and analysis, writing of initial manuscript, manuscript revision*: Jacob Oeding. *Manuscript revision*: Kyle Kunze, Ayoosh Pareek, Kristian Samuelsson.

## CONFLICT OF INTEREST STATEMENT

The authors declare no conflicts of interest.

## ETHICS STATEMENT

This study was exempt from institutional review board approval.

## Data Availability

The data that support the findings of this study are available from the corresponding author upon reasonable request.
